# Comparative Study of Subcutaneous and Orthotopic Mouse Models of Prostate Cancer: Vascular Perfusion, Vasculature Density, Hypoxic Burden and BB2r-Targeting Efficacy

**DOI:** 10.1038/s41598-019-47308-z

**Published:** 2019-07-31

**Authors:** Wenting Zhang, Wei Fan, Satyanarayana Rachagani, Zhengyuan Zhou, Subodh M. Lele, Surinder K. Batra, Jered C. Garrison

**Affiliations:** 1 Department of Pharmaceutical Sciences, University of Nebraska Medical Center, Omaha, NE USA; 2 Center for Drug Delivery and Nanomedicine, University of Nebraska Medical Center, Omaha, NE USA; 3 Eppley Cancer Center, University of Nebraska Medical Center, Omaha, NE USA; 4 Department of Biochemistry and Molecular Biology, University of Nebraska Medical Center, Omaha, NE USA; 5 Department of Radiology, Duke University Medical Center, Durham, NC USA; 6 Department of Pathology and Microbiology, University of Nebraska Medical Center, Omaha, NE USA

**Keywords:** Drug delivery, Cancer models, Cancer microenvironment

## Abstract

The gastrin-releasing peptide receptor (BB2r) is overexpressed in a variety of cancers including prostate cancer. As a consequence, the development of BB2r-targeted diagnostic/therapeutic radiopharmaceuticals has been widely explored. Both subcutaneous and orthotopic mouse models have been extensively used in BB2r-targeted agent development, but side-by-side studies examining how biological parameters (tumor perfusion efficacy, hypoxic burden and microvasculature density) impact BB2r-targeted agent delivery has not been reported. Herein, we examine these biological parameters using subcutaneous and orthotopic PC-3 xenografts. Using a dual isotope biodistribution study, tumor perfusion was accessed using [^99m^Tc]NaTcO_4_ and BB2r-targeted uptake evaluated by utilization of a novel ^177^Lu-labeled conjugate ([^177^Lu]Lu-DOTA-SP714). Immunofluorescence, immunohistochemistry and autoradiography were utilized to examine the tumor vascular density, hypoxic burden and microdistribution of the BB2r-targeted agent. Our studies demonstrated that compared to the subcutaneous model the PC-3 orthotopic tumors had significantly higher levels of perfusion that led to higher BB2r-targeted uptake and lower levels of hypoxia burden. It is anticipated that our results will allow researchers to better understand the biological variables affecting drug delivery and assist them in more clearly interpreting their results in this common prostate cancer mouse model.

## Introduction

The gastrin-releasing peptide receptor (BB2r) is a G-protein coupled receptor that has been of significant interest to the field of cancer drug development due to its overexpression in a variety of cancers, including prostate cancer^1^. Accordingly, numerous theranostic BB2r-targeted agents have been developed over the years with several agents going on to clinical trials^2–6^. Generally, these agents have been based on synthetic derivatives of the C-terminal portions of the bombesin (BBN) peptide, which retains nanomolar affinities for the BB2r. To evaluate newly developed BB2r-targeted agents, researchers have numerous tumor models (e.g., subcutaneous, orthotopic and genetically engineered mouse models) from which to choose^7^. How well these models mirror the pathology and clinical chemotherapeutic response of human cancers is generally inversely related to the technical difficulties in generating the model. As such, researchers typically rely on relatively simple subcutaneous tumor xenografts to first study the biological performance of new agents. These models are invaluable for examining the pharmacokinetic profiles of agents, verifying *in vivo* BB2r-targeting and identifying lead compounds. Yet, it is well-known that the tumor biology of subcutaneous xenografts is not as reflective as other models (e.g., orthotopic) of human tumors^8^. Arguably the most common xenograft model to evaluate BB2r-targeted agents is the murine xenograft model utilizing the human prostate cancer PC-3 cell line, which has been shown to highly overexpress the BB2r^9–12^. Previous reports have compared BB2r-targeted uptake in subcutaneous and orthotopic PC-3 mouse models^13^, but a thorough investigation into how the tumor biology of the models affect BB2r-targeted agent delivery remains unreported.

The tumor microenvironment (TME) is a wide-ranging term utilized to describe the complex environment in which tumor exist^14^. In regard to drug delivery, perhaps the most important TME variable is the density and functionality of the tumor vasculature. Tumor perfusion, essentially how well the tumor vasculature distributes blood throughout the tumor, directly influences the delivery of drugs and biologically important nutrients (e.g., oxygen). As a consequence, many solid tumors, including prostate cancer, contain significant regions of hypoxia due to their distorted and insufficiently developed vasculature^15^. Hypoxia is also recognized as a crucial stimulus for tumor angiogenesis, metastasis and, in many cases, aggressiveness^16^.

**Figure 1:**
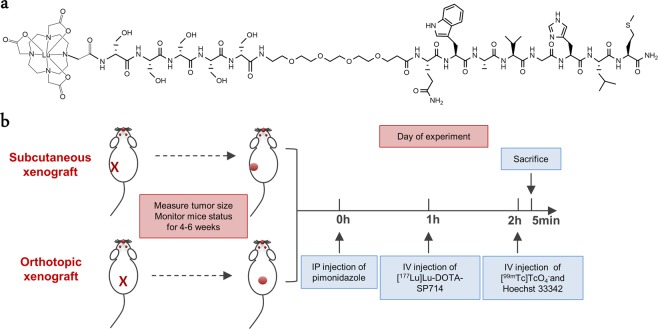
(**a**) Structure of [^177^Lu]Lu-DOTA-SP714. (**b**) Experimental design and timeline.

The purpose of this study is to elucidate the impact of tumor perfusion and vascular density on BB2r-targeted drug delivery and tumor hypoxia. Specifically, we undertake this investigation in subcutaneous and orthotopic PC-3 xenograft mouse models over a spectrum of tumor volumes to explore similarities and differences between the two models. By this study, we hope to give the research community utilizing these models a better understanding of the cause-effect relationship between these variables and their potential impact on drug delivery. To this end, sodium pertechnetate ([^99m^Tc]NaTcO_4_), a blood perfusion agent^17–22^, was employed to evaluate the PC-3 tumor perfusion efficiency. The functional vasculature density of the tumors was measured using Hoechst staining^23^. A novel BB2r-targeted analog, DOTA-(D)S-(D)S-(D)S-(D)S-(D)S-PEG_3_–BBN(7–14)NH_2_ (DOTA-SP714), was utilized to examine *in vivo* BB2r uptake and tumor distribution (Fig. 1a). Lastly, the hypoxic burden of the PC-3 tumors was measured by pimonidazole staining^24^.

## Results

### Synthesis and radiolabeling of DOTA-SP714

DOTA-SP714 was synthesized with a yield of 26.6% as determined by HPLC-MS. The peptide was labeled with LuCl_3_ or [^177^Lu]LuCl_3_ and subsequently purified by RP-HPLC. Analysis of the chromatograms revealed the purity of the radiolabeled peptide was ≥95% with a radiolabeling yield of 90.5% (Table 1).

**Table 1:** Characterization of Conjugate.

Analogue	Molecular Formula	MW (m/z, [M+H]^+^)		RP-HPLC Retention Time/min	IC_50_/nM^†^	LogD (pH_7.4_)^‡^
		Calcd	Found			
DOTA-SP714	C_85_H_137_N_23_O_31_S	2008.0	2008.6	8.67	9.8 ± 1.7	—
Lu-DOTA-SP714	C_85_H_134_LuN_23_O_31_S	2179.9	2179.9	8.65	1.0 ± 1.7	−2.2 ± 0.1

^†^Values represent mean ± SEM (n = 6).

^‡^Values represent mean ± SD (n = 3).

### Distribution coefficient and competitive binding studies

The distribution coefficient (mean ± SD) at pH = 7.4 (LogD_7.4_) for [^177^Lu]Lu-DOTA-SP714 was determined to be −2.2 ± 0.1 by radiometric analysis. The hydrophilic nature of the radioconjugate suggests that cellular internalization should occur only through BB2r-mediated endocytosis. Subsequently, competitive binding studies demonstrated that both peptides demonstrated low nanomolar binding affinity, with ^nat^Lu-DOTA-SP714 (1.0 ± 1.7 nM) giving a significantly higher affinity relative to the unlabeled conjugate (9.8 ± 1.7 nM). Both the IC_50_ and the LogD_7.4_ values are provided in Table 1.

### Radiochemical stability studies

To investigate the susceptibility of [^177^Lu]Lu-DOTA-SP714 to radiolytic degradation and examine the efficacy of stabilizers, two stabilizing buffers formulated using ascorbic acid with/without selenomethionine were tested and compared to a control PBS buffer. The results of these studies are depicted in Figure S2. In PBS alone, 39.9% of a 0.37 MBq/mL (100 µCi/mL) solution of [^177^Lu]Lu-DOTA-SP714 degraded over the course of 72 h. As expected, the addition of ascorbic acid (40 mg/mL) demonstrated substantial improvements in stability with only 17.3% radiolytic breakdown. However, the combination of ascorbic acid (40 mg/mL) and selenomethionine (0.2 mg/mL) gave superior results with only 5.3% breakdown observed by the 72 h time point.

### Animal model establishment

In this study, subcutaneous and orthotopic xenograft models were generated. For the subcutaneous model, the diameters of the tumors were determined by caliper measurements, while tumor growth in the orthotopic model was monitored weekly after implantation surgery using bioluminescence imaging (Fig. S3a,b). The orthotopic model gave a gradual increase in estimated tumor volumes over the 6-weeks, while the subcutaneous model showed a sharp and rapid increase in tumor size starting at 4^th^-week (Fig. S3c). While the above measurements were used to non-invasively monitor tumor growth, the assignment of the tumor volume in our subsequent studies is based on caliper measurements of the excised tumors. For both measurements, tumor volumes were calculated by using the following formula: Volume = (Length × Width^2^)/2.

### BB2r-Targeted peptide uptake and perfusion in PC-3 tumor xenograft models

**Figure 2:**
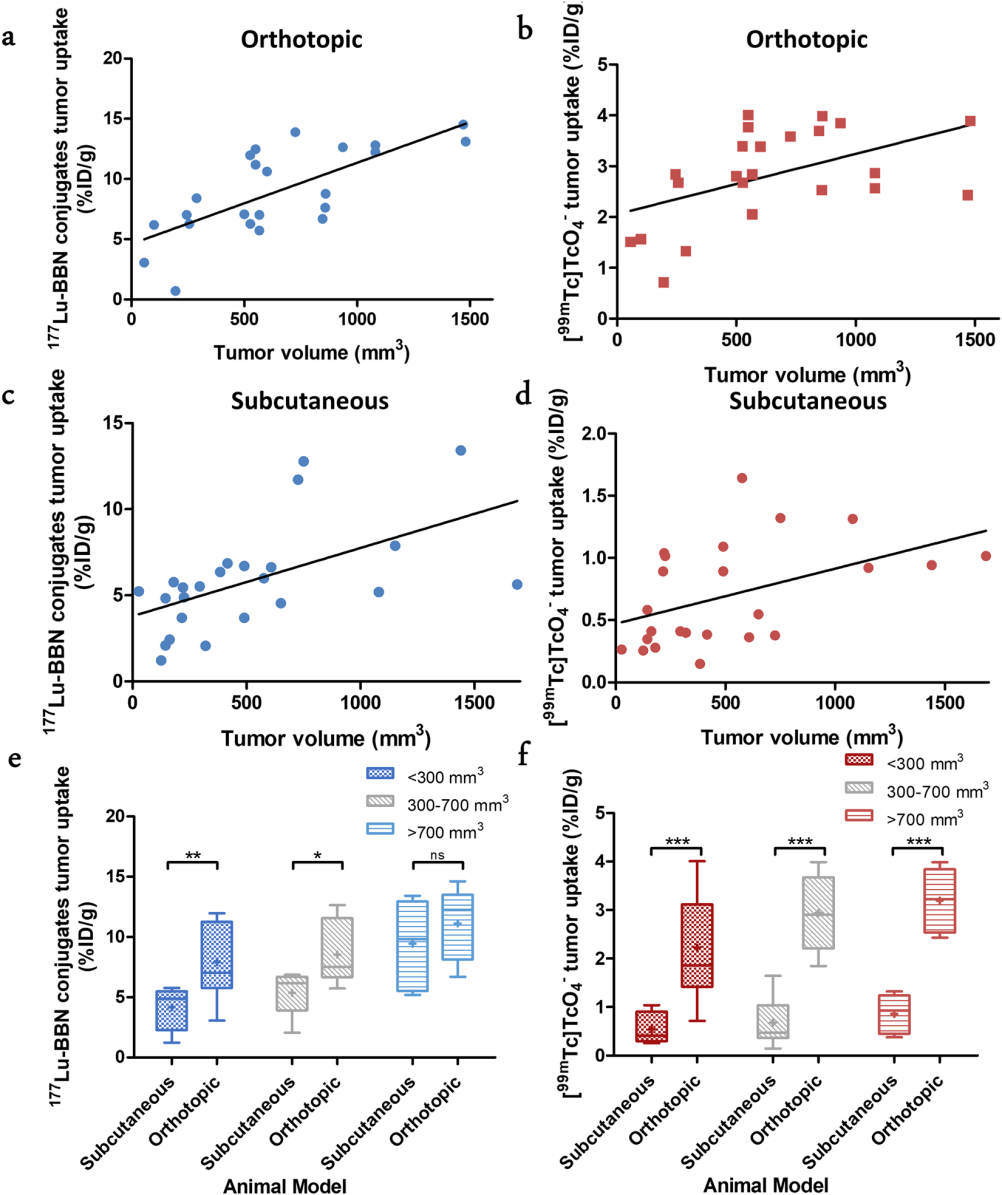
Correlation of tumor uptake of [^177^Lu]Lu-DOTA-SP714 (**a**,**c**) and of [^99m^Tc]TcO_4_^−^ (**b**,**d**) in orthotopic (n = 23) and subcutaneous (n = 24) model respectively. (**e**,**f**). Grouped box-whisker plot of tumor uptake of [^177^Lu]Lu-DOTA-SP714 and [^99m^Tc]TcO_4_^−^ in two animal models. (*p < 0.05, **p < 0.01, ***p < 0.001, ns = no significance, +: mean, line at median).

To examine the *in vivo* BB2r-targeting efficacy and tumor perfusion of the two mouse models, we performed dual, simultaneous biodistribution studies utilizing the [^177^Lu]Lu-DOTA-SP714 and [^99m^Tc]NaTcO_4_. The %ID/g in each organ and the total excretion (%ID) of the radioactivity from the mice are presented in Table S1. Overall, the scatter plots of the tumor uptake of [^177^Lu]Lu-DOTA-SP714 and [^99m^Tc]TcO_4_^−^ against tumor volume in both animal models are depicted in Fig. 2a–d. There was a significant moderate to strong positive correlation (Table 2), observed between tumor volume and BB2r-uptake (%ID/g) of the [^177^Lu]Lu-DOTA-SP714 (r = 0.55, p = 0.0049 and r = 0.73, p = 0.0001) as well as tumor perfusion ([^99m^Tc]TcO_4_^−^) (r = 0.47, p = 0.021 and r = 0.49, p = 0.017) in subcutaneous and orthotopic mice models, respectively. Interestingly, the correlation (Figure S4) between tumor perfusion ([^99m^Tc]NaTcO_4_) and BB2r-uptake ([^177^Lu]Lu-DOTA-SP714) was weak to moderate for the subcutaneous (r = 0.28, p = 0.19) and orthotopic (r = 0.66, p = 0.0006) models, respectively, suggesting tumor size is a better indicator of BB2r-uptake in both models.

**Table 2:** Pearson Correlation Coefficient and p-Value of Tumor Volume with Uptake of Radiotracer in Mice Models.

Tumor uptake (%ID/g)		Tumor volume (mm^3^)	
		Pearson r	p-value
**Orthotopic**	[^177^Lu]Lu-DOTA-SP714	0.73	0.0001
	[^99m^Tc]TcO_4_^−^	0.49	0.017
**Subcutaneous**	[^177^Lu]Lu-DOTA-SP714	0.55	0.0049
	[^99m^Tc]TcO_4_^−^	0.47	0.021

Grouping tumor volumes categorically, <300, 300–700 and >700 mm^3^, revealed some interesting trends (Fig. 2e,f). For the orthotopic model, the mean (mean ± SD) categorical tumor volumes were 215.62 ± 103.90, 574.14 ± 73.89 and 1052.78 ± 256.12 mm^3^ for <300, 300–700 and >700 mm^3^ groups respectively. With respect to the subcutaneous model, the corresponding mean categorical tumor volumes for <300, 300–700 and >700 mm^3^ groups were 169.06 ± 74.94, 491.69 ± 114.89 and 1139.25 ± 378.68 mm^3^. In the orthotopic model, the average uptake (mean ± SD) of the [^177^Lu]Lu-DOTA-SP714 was 8.07 ± 3.23, 8.55 ± 2.65 and 11.13 ± 2.88%ID/g for the <300, 300–700 and >700 mm^3^ groups, respectively. By the ANOVA test, while close (p = 0.061), this trend of increased BB2r-uptake with increasing tumor volume did not meet statistical significance given our study size. For the subcutaneous model, the mean uptake of the radioconjugate increased significantly (p = 0.0014) with increasing tumor size from 4.16 ± 1.74 (<300 mm^3^) to 5.35 ± 1.75 (300–700 mm^3^) to 9.44 ± 3.66 (>700 mm^3^) %ID/g. Uptake of the perfusion agent in subcutaneous tumors increased from 0.54 ± 0.32 (<300 mm_3_) to 0.68 ± 0.49 (300–700 mm^3^) to 0.86 ± 0.37 (>700 mm^3^) %ID/g as tumor volume increased (p = 0.15). Likewise, the average tumor uptake of the [^99m^Tc]TcO_4_^−^ for the orthotopic model increased when comparing the <300 mm^3^ (2.23 ± 1.09%ID/g), 300–700 mm^3^ (2.94 ± 0.76%ID/g) and >700 mm^3^ (3.19 ± 0.66%ID/g) groups (p = 0.078).

The most striking differences were observed when comparing the BB2r-mediated uptake and perfusion between models at each tumor volume category. The average uptake of the [^177^Lu]Lu-DOTA-SP714 for the orthotopic model was 1.9, 1.6 and 1.2-fold higher compared to the respective tumor volumes of the subcutaneous model (<300 mm^3^, p = 0.0055; 300–700 mm^3^, p = 0.011; and >700 mm^3^ groups, p = 0.34). Similarly, a substantial and statistically significant increase in tumor perfusion was observed for the orthotopic versus the subcutaneous model for each tumor volume category. The average uptake of perfusion tracer in the orthotopic model was 4.1, 4.3 and 3.7-fold higher compared to the respective subcutaneous model (<300 mm^3^, p = 0.0008; 300–700 mm^3^, p = 0.0001; and >700 mm^3^, p = 0.0001). Based on the examination of the biodistribution data, this large difference in perfusion between models may well be overstated due to a decreased clearance rate of the radiotracer from the orthotopic model. Biodistribution data (Table S1) show that the blood retention for the orthotopic model was 1.5–3.1 times higher compared to the subcutaneous model. Furthermore, this trend was reflected in the well-known stomach uptake of ^99m^Tc-pertechnetate which was approximately 8-fold higher for the orthotopic model^25^. These data suggest the orthotopic model has a lower rate of blood clearance for ^99m^Tc-pertechnetate. Speculatively, this may be due to the placement of the orthotopic tumor restricting renal excretion^26^ and reducing ^99m^Tc-pertechnetate clearance from the body. If so, this effect was asymmetric since the blood retention values for the BB2r-targeted agent ([^177^Lu]Lu-DOTA-SP714) between the two models were not statistically different.

### Quantification of hypoxic burden and blood vessel density in PC-3 tumors

Prior to the sacrifice of the mice, pimonidazole (hypoxia marker) and Hoechst (functional vasculature marker), were administered to examine the hypoxia burden and vascular density of the human PC-3 tumor xenografts. The excised human PC-3 tumors were sectioned and histologically evaluated for fluorescence signals using adjacent tumor slices. Both signals were calculated by mean intensity per µm^2^ and grouped by tumor volume groups for each mouse model (Table 3).

**Table 3:** Mean Intensity/µm^2^ of Hypoxic Burden and Blood Vessel Density in Tumor Xenograft.

Tumor volume (mm^3^)	Mean Intensity/µm^2^ (mean ± SEM)^*^			
	Subcutaneous		Orthotopic	
	Hypoxic burden	Vasculature Density	Hypoxic burden	Vasculature Density
**<300**	9.47e-6 ± 1.74e-6	4.06e-6 ± 8.54e-7	4.33e-6 ± 1.41e-6	8.33e-6 ± 1.42e-6
**300–700**	3.84e-5 ± 6.98e-6	9.90e-6 ± 1.88e-6	1.18e-5 ± 2.64e-6	8.67e-6 ± 6.44e-7
**>700**	2.69e-5 ± 2.98e-6	2.11e-5 ± 4.30e-6	5.75e-6 ± 1.45e-6	1.39e-5 ± 1.81e-6

*Values represent mean ± SEM (n ≥ 6).

**Figure 3:**
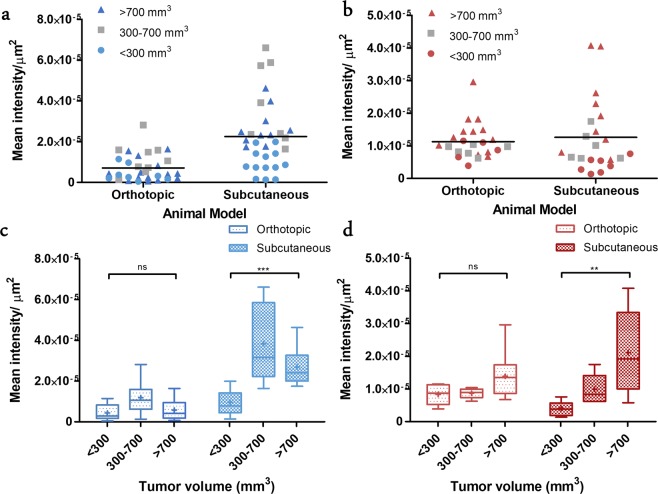
Summary of quantification of (**a**) hypoxia burden (**b**) blood vessel density of tumor slides in two mouse models. Line at grand mean. Box-whisker plots are indicating the variance of (**c**) hypoxia burden and (**d**) blood vessel density between different tumor volume groups in two animal models. (+: mean, line at median).

On average, subcutaneous tumors exhibited a 3.4-fold higher level of hypoxia burden (signal) than the orthotopic tumors (Fig. 3a). For both xenograft models, the tumors demonstrated a trend toward maximal hypoxia burden for the 300–700 mm^3^ group (Fig. 3c). Overall, there was no significant difference (p = 0.13) in the hypoxia burden among the tumor volume groups in the orthotopic model (one-way ANOVA). However, a significant difference in hypoxia levels was observed within the tumor volume groups of the subcutaneous mouse model (p < 0.001). A comparison of hypoxia burden for the <300 mm^3^ tumor groups revealed no significant difference (p = 0.053) between models. In contrast, significant differences (p = 0.002 and p < 0.0001, correspondingly) between xenograft models were observed for the 300–700 and >700 mm^3^ groups, largely due to the substantial increase in hypoxia levels in the subcutaneous models for tumor volumes exceeding 300 mm^3^. Specifically, the hypoxia burden increased 4.0-fold in the subcutaneous model for the 300–700 mm^3^ group compared to the <300 mm^3^ group (p < 0.001). In both tumor models, the hypoxia levels decreased on average by 30–50% when the tumor size exceeded 700 mm^3^ compared to the respective 300–700 mm^3^ group (p > 0.05).

The functional tumor blood vessel density of the excised PC-3 tumors are depicted in Fig. 3b. On average, the subcutaneous tumors had a slightly higher functional vascular density compared to the orthotopic tumors. For both xenograft models, there was a trend toward higher vascular density as tumor volume increased (Fig. 3d). For the subcutaneous model, there was a 2.4 and 5.2-fold increase of functional vascular density in the 300–700 (p = 0.013) and >700 mm^3^ groups (p = 0.0041) relative to the <300 mm^3^ group. With respect to the orthotopic tumors, no increase in vascular density was observed for the 300–700 mm^3^ (p = 0.82) group compared to the <300 mm^3^ group. Although, a 1.7-fold increase in vessel density was observed for the >700 mm^3^ (p = 0.084) tumor volume group relative to the smaller groups. For tumor group comparisons between models, the orthotopic tumors of the <300 mm^3^ group had a significantly (p = 0.0021) higher vascular density compared to the subcutaneous model, but no statistically significant differences were found between the 300–700 and >700 mm^3^ groups (p = 0.55 and p = 0.11, correspondingly).

### Qualitative examination of the distribution of the BB2r-Targeted peptide in tumor xenografts

**Figure 4:**
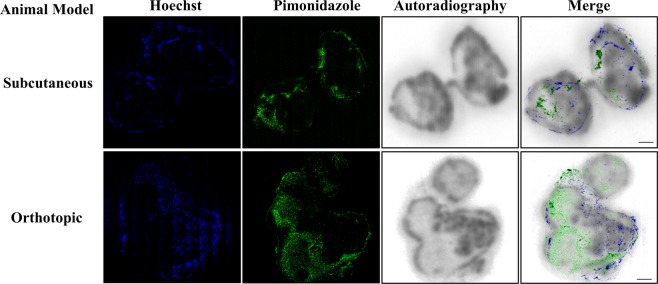
Representative confocal microscopy images of adjacent tumor slides stained with Hoechst 33342 (blue) and anti-pimonidazole-FITC (green) and performed autoradiography respectively in two mouse models. Scale bar: 1 mm.

To better examine the distribution of [^177^Lu]Lu-DOTA-SP714 in the two xenografts models in relation to the hypoxic fractions and functional vasculature of tumors, sections of tumors were examined by autoradiography (BB2r-targeted peptide) and confocal microscopy to determine the hypoxic areas (Green) and functional vasculature (Blue). The representative images from the 300–700 mm^3^ group are shown in Fig. 4. As expected, the predominant localization of the [^177^Lu]Lu-DOTA-SP714 was in areas that had significant functional vasculature. While in some areas that lacked vasculature were hypoxic and exhibited a significantly lower concentration of [^177^Lu]Lu-DOTA-SP714.

### H&E staining of non-target tissues

**Figure 5:**
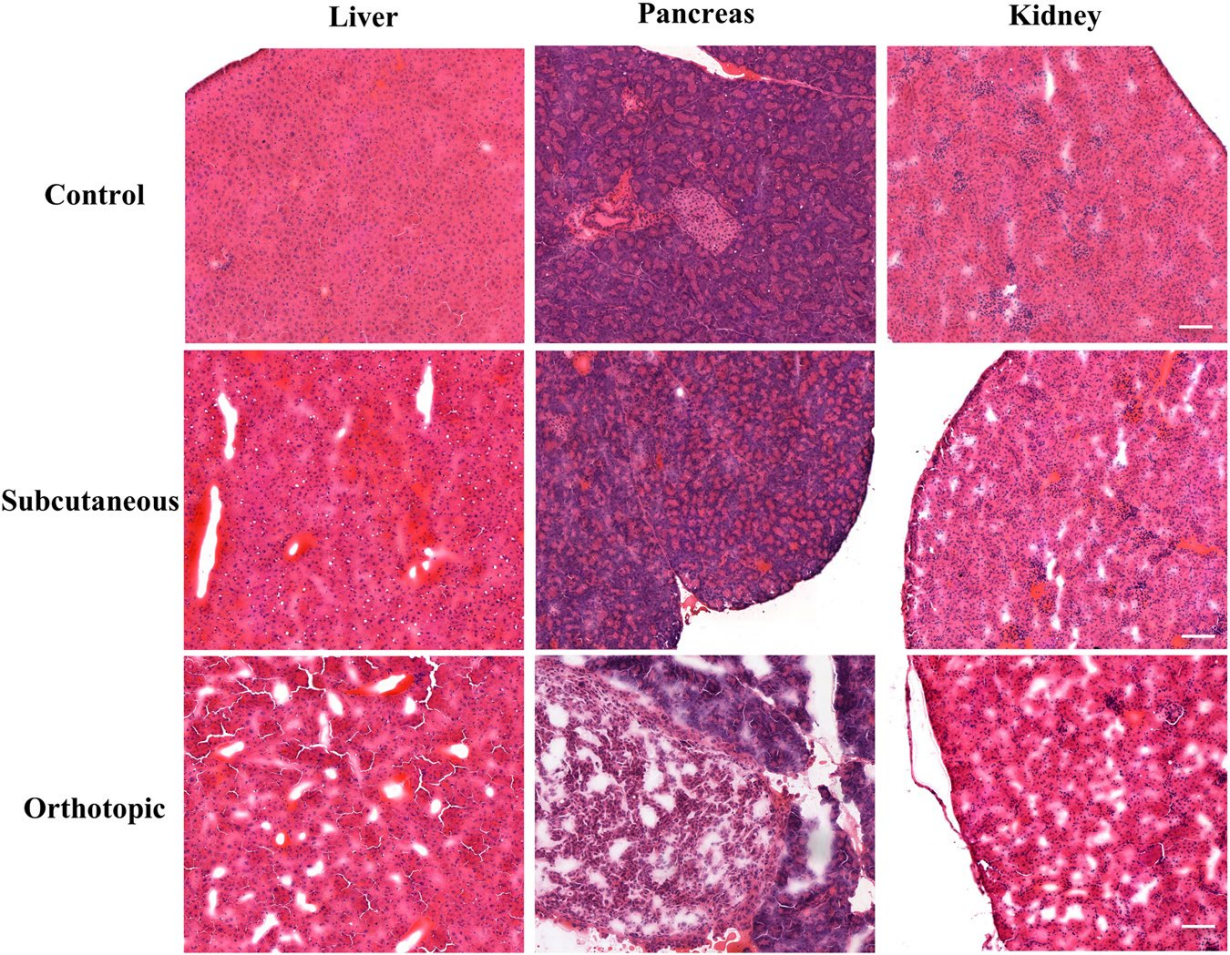
Representative H&E image of the liver, pancreas and kidney tissue sections (10×) of non-tumor bearing, subcutaneous and orthotopic xenograft mouse respectively. Scale bar: 100 µm.

Upon dissection of the mice from the orthotopic mouse model with tumors exceeding 700 mm^3^, gross anatomy abnormalities of the kidneys, pancreas and liver were perceived. The kidneys were often found to be pale and enlarged and the formation of fluid-filled cysts on the surface of the kidneys was observed, likely due to hydronephrosis. In several cases, enlargement and/or discoloration of the liver and pancreas were also noted. The tissues taken from orthotopic models along with an analogous subcutaneous model (>700 mm^3^ group) and normal control mouse were sectioned and underwent H&E staining, see Fig. 5. All of the sectioned tissues associated with the orthotopic model were more diffuse than subcutaneous and normal controls probably due to the increased interstitial volume resulting from reduced urine output. For the orthotopic tumor group, micrometastases were observed in the pancreas, but, surprisingly, were not found in the liver or kidney sections. No micrometastases were observed in the sections obtained from the subcutaneous model.

## Discussion

The purpose of this work is to investigate how the vascular density and perfusion of tumors impact the hypoxia burden and drug delivery of BB2r-targeted agents. Specifically, we investigated these factors in subcutaneous and orthotopic xenografts across a range of tumor volumes. By understanding this, we hope to provide researchers a better understanding of these biological variables as well as the advantages and limitations of both subcutaneous and orthotopic models for BB2r-targeted agent development.

We first synthesized and characterized a new hydrophilic, BB2r-targeted radioconjugate ([^177^Lu]Lu-DOTA-SP714). In addition to examining it’s chemical and *in vitro* properties, we also accessed the solution stability of the radioconjugate. This investigation was prompted out of the need to perform *in vivo* experiments with large numbers of mice and the desire to reduce the need for the constant synthesis of the radioconjugate. It is well established that ionizing radiation (e.g., α^2+^ and β^−^ particles) can directly or indirectly, through the generation of solvated electrons and free radicals species, degrade peptides and proteins^27^. Ascorbic acid and selenomethionine have been demonstrated to have a protective effect against radiolytic degradation in both preclinical and clinical studies^28–30^. Indeed, Chen and co-workers examined these radioprotectants among others with [^177^Lu]Lu-AMBA, a clinically investigated BB2r-targeted agent^31^. Similar to their findings, our studies revealed that the combination of the two radioprotectants, ascorbic acid (40 mg/mL) and selenomethionine (0.2 mg/mL), was better than ascorbic acid alone with approximately 95% of the radioconjugate intact by 72 h.

The establishment of the subcutaneous and orthotopic PC-3 xenograft model was carried out over a four to eight-week period depending on the model and the desired tumor size. The measured tumor size for orthotopic tumors initially outpaced subcutaneous tumors, but the subcutaneous tumors demonstrated a more rapid growth at approximately 4-weeks post-inoculation. The disparity of growth rates between two models likely stem from the different measurement techniques employed and the initial cell numbers administered. Based on body weight measurements, the overall health of the mice appeared good across models and tumor size ranges investigated (Figure S5). However, for the largest orthotopic tumor volume group (>700 mm^3^), abnormalities were found upon gross examination of the kidneys, liver and pancreas. This prompted the histological examination of these tissues for both models. The tissues obtained from the orthotopic model were more diffuse and were consistent with larger interstitial water content, likely due to urinary obstruction resulting from the PC-3 tumor in the prostate. This observed condition has been noted for other orthotopic prostate cancer mouse models^32^.

The examination of the perfusion characteristics of the two models revealed some interesting findings. The average perfusion of the tumors increased as tumor size increased. However, the orthotopic model showed substantially higher tumor perfusion (3.7–4.3 fold) compared to the subcutaneous model. This is interesting given the similar amount of functional vasculature in both models based on Hoechst staining. This comparably higher perfusion rate has also been observed in other orthotopic relative to subcutaneous models^33–35^. A factor that may be contributing to the lower perfusion of subcutaneous tumors is a higher interstitial fluid pressure (IFP). In general, the functional vasculature of the orthotopic tumors is more spatially distributed and have larger vessel diameters relative to subcutaneous tumors^36,37^. These factors have been shown to reduce the level of IFP. With that said, as we discussed earlier, the perfusion agent (^99m^Tc-pertechnetate) exhibited a lower rate of renal clearance possibly overstating the magnitude in the differences in perfusion between the two models. Careful consideration should be given regarding the blood and renal clearance of the radiotracer when comparing tumor uptake values.

The BB2r-targeting efficacy of the [^177^Lu]Lu-DOTA-SP714 was examined in both PC-3 xenograft models. Generally, the average %ID/g uptake of the radioconjugate increased as the tumor size increased. Specifically, orthotopic tumors exhibited significantly higher uptake compared to subcutaneous tumors across tumor groups. This is almost certainly due to the increased perfusion of orthotopic over subcutaneous tumors.

Using pimonidazole, the hypoxia burden of the tumors was evaluated for both models. For the orthotopic tumors, no significant differences in hypoxia levels were observed across the tumor volume ranges. However, the subcutaneous tumors exhibited significant increases in hypoxia with tumor sizes that exceeded 300 mm^3^. The increased hypoxia levels for the 300–700 and >700 mm^3^ tumor groups may be attributable to the spatial distribution of the functional vascular on the periphery of tumors. For small tumors, the peripheral vasculature can adequately perfuse the whole tumor. However, as the tumor grows vasculature may become incapable of perfusing the center of the tumor resulting in significant increases in hypoxia burden^38^ and tumor necroses^39–42^. While hypoxia was observed for both models, medium to large subcutaneous tumors more reliably gave tumors with significant fractions of hypoxia. This phenomenon has been observed by other researchers as well^43,44^. In this particular study, we did not examine the extent of tumor necroses and its correlation to PC-3 tumor volumes to determine its impact, if any, on BB2r-targeted agent delivery. This is certainly an interesting question and something we may examine in our future work. Overall, these findings suggest PC-3 tumors >300 mm^3^ may be a more appropriate volume to investigate the efficacy of hypoxia-targeted or hypoxia-selective agents.

## Conclusion

Orthotopic models are known to better simulate clinical prostate cancer, particularly with respect to the tumor microenvironment, compared to subcutaneous models. To better understand how the biology of these tumor models’ impact BB2r-targeted agent delivery, we examined the tumor vascular perfusion, microvasculature density and hypoxia burden of orthotopic and subcutaneous PC-3 xenograft mouse models. Compared to the subcutaneous model, the results demonstrate that the orthotopic PC-3 tumors have higher vascular perfusion that leads to higher BB2r targeting as well as a lower hypoxic burden. While the vessel density was slightly lower in the orthotopic model compared to subcutaneous ones, no statistical significance was observed. In general, for both models, BB2r-targeting, perfusion and vascular density increased with increasing tumor volume. Immunofluorescence and autoradiography illustrated the microdistribution pattern of the BB2r-targeted conjugate relative to functional vasculature and hypoxic regions. As expected, higher concentrations of the radiolabeled conjugate were observed near functional vasculature compared to hypoxic regions that were devoid of functional vasculature. Overall, this work demonstrates that the tumor microenvironments of orthotopic and subcutaneous PC-3 tumors are impactfully different in terms of drug delivery. Careful consideration should be taken when comparing the data of BB2r-targeted agents, as well as other targeted agents, in orthotopic and subcutaneous tumor models.

## Materials and Methods

Full details regarding the materials and equipment are presented in the supplemental materials. Also given in the supporting information is our methodology concerning peptide synthesis, cell culture, luciferase transfection, distribution coefficient studies and competitive binding studies.

### Radiochemical stability studies

The radiochemical stability of [^177^Lu]Lu-DOTA-SP714 was evaluated by RP-HPLC using various stabilizing buffers. Briefly, 0.37 MBq of radiolabeled compounds were incubated with 1 mL of: A) PBS; B) ascorbic acid in 0.9% sodium chloride solution (40 mg/ml); and C) Selenomethionine (0.2 mg/ml) and ascorbic acid (40 mg/ml) in 0.9% sodium chloride solution at 4 °C for 24, 48 and 72 h. The stability of the radioconjugate at each time point was calculated based on the integration of the RP-HPLC chromatograms.

### Animal models

All animal experiments were performed in accordance with the NIH animal use guideline and protocol approved by the Institutional Animal Care and Use Committee (IACUC) at the UNMC. NOD.CB17-*Prkdc*^sicd^*I*J (NOD SCID) mice were purchased from the Jackson Laboratory (Bar Harbor, ME). The mice were housed in groups of five in the UNMC animal facility for the entire tumor generation period. All mice were under constant temperature (set up at 21 °C) and humidity on a 12-hour light/dark cycle, which lights on at 7:00 am. Standard food and filtered water were available *ad libitum*.

For the subcutaneous model^9^, 5-week old female SCID mice were inoculated in the flanks with 5 × 10^6^ PC-3 cells (American Type Culture Collection (ATCC), VA) in Matrigel^®^. The tumor size was monitored by caliper. For the generation of the orthotopic model^45^, the PC-3-Luc cells were selected by 0.5 µg/mL puromycin twice before inoculation. The male 6-weeks SCID mice were anesthetized, the muscles of the abdomen area were cut after retracting the skin, and the prostate gland exposed. 50 μL of PC-3-Luc cell suspension at 0.5 × 10^6^/mL in Matrigel^®^ was injected into a dorsal prostatic lobe. The wound was closed in two layers and the skin was clipped. Animals were given analgesic drugs for 3 days and the tumors were monitored by an IVIS optical imaging system.

### Bioluminescent imaging

For *in vitro* imaging, PC-3-Luc cells (30,000 cells/well) were diluted and plated in a 96-well plate. D-luciferin (50 μL, 150 μg/mL) was added to the media five min prior to imaging (Figure S1). For *in vivo* imaging, mice were given D-luciferin (100 μL, 15 mg/mL) 15–20 min prior to anesthetization by isoflurane. At imaging, the mice were transferred to the IVIS enclosure and images were acquired by IVIS^®^ Spectrum software with auto-exposure. Regions of interest from each image were selected and quantified.

### *In Vivo* biodistribution studies

The mice were injected, monitored and dissected in the lab with the temperature of 23 °C and 17% relative humidity. All animals were awake until sacrifice. The body weight, tumor volume and tumor luminescence of the mice were recorded every three days. At four-to-seven weeks post-xenograft implantation, the mice were divided into three groups by tumor volume: <300, 300–700 and >700 mm^3^ in both animal models. Tumor volume groups of the subcutaneous models had an N = 8 (<300 mm^3^), 9 (300–700 mm^3^) and 9 (>700 mm^3^) of mice separately. The orthotopic tumor volume groups had an N = 9 (<300 mm^3^), 8 (300–700 mm^3^), and 6 (>700 mm^3^) of mice separately. Each mouse (average weight: 20 g for female mice and 25 g for male mice) was treated with pimonidazole solution (80 mg/kg in PBS) via intraperitoneal injection. After 1 h, the mice received an intravenous injection of 10 µCi (370 kBq) of the radio-RP-HPLC peak purified ^177^Lu-labeled conjugate ([^177^Lu]Lu-DOTA-SP714) in 100 μL of PBS. After an additional 1 h, the mice were injected intravenously with a PBS solution containing 10 µCi (370 kBq) of [^99m^Tc]NaTcO4 and 15 mg/kg Hoechst 33342. The animals were sacrificed 5 minutes later, and their tissues collected. The excised tissues were weighed, the radioactivity for each tissue was measured by γ-counter and the percentage injected dose per gram (%ID/g) was calculated for each tissue.

### Microscopy and autoradiography

At the end of the biodistribution studies, the tumor, liver, kidney and pancreas from the mice were rinsed by deionized water, dried and embedded by O.C.T compound on dry ice. The adjacent cryostat tumor slides (10 µm) were scanned for Hoechst 33342 and anti-pimonidazole-FITC by confocal microscopy and exposed to a storage phosphor screen to be scanned by the Typhoon imaging system using 25 µm resolution. The fluorescent intensity of images was measured by ZEN software (blue edition). The H&E staining sections of the liver, kidney and pancreas were evaluated by a clinical pathologist. Frozen slides have significant high background fluorescence caused by non-seal storage were excluded from the analysis.

### Statistical analysis

Data were presented as Mean ± SD/SEM as noted. IC50 values were determined by nonlinear regression using the one-binding-site model of GraphPad PRISM 5. Pearson correlation coefficients (r) were calculated to assess the relationship between tumor volume and tumor uptake of the radiotracer ([^177^Lu]Lu-DOTA-SP714 and [^99m^Tc]NaTcO_4_). *In vitro* stability studies of the radioconjugate in various buffers were analyzed by the two-tailed Student’s t-test. For comparison between the two models, analysis of the biodistribution studies, hypoxic burden and blood vessel density of tumor was carried out by a two-tailed Student’s t-test. Comparisons of the means of the BB2r-uptake, blood perfusion, hypoxic burden and blood vessel density of the tumors among the three tumor volume groups for each animal model were analyzed by one-way ANOVA. If the overall p-value was statistically significant, pairwise comparisons were adjusted using Tukey’s method. A p-value < 0.05 was considered statistically significant.

### Ethical approval

All animal experiments were performed in accordance with the NIH animal use guideline and protocol approved by the Institutional Animal Care and Use Committee (IACUC) at the University of Nebraska Medical Center. None human participants were involved in this study.

## Supplementary information

Supplementary information

## Data Availability

The datasets generated during and/or analyzed during the current study are available from the corresponding author on reasonable request.
