# Supplementary material for: Comparative Study of Subcutaneous and Orthotopic Mouse Models of Prostate Cancer: Vascular Perfusion, Vasculature Density, Hypoxic Burden and BB2r-Targeting Efficacy
Source: Sci Rep. 2019 Jul 31;9:11117. doi: 10.1038/s41598-019-47308-z (PMC6668441; doi:10.1038/s41598-019-47308-z)
Supplement: Supplementary file 1 — Supplementary information [file 41598_2019_47308_MOESM1_ESM.pdf]

Supplementary Information

Comparative Study of Subcutaneous and Orthotopic Mouse Models of Prostate Cancer: Vascular Perfusion Efficiency, Vasculature Density, Hypoxia Burden and BB2r-Targeting Efficacy.

Wenting Zhang[1,2], Wei Fan[1,2], Satyanarayana Rachagani[4], Zhengyuan Zhou[6], Subodh M. Lele[5], Surinder K. Batra[4], Jered C. Garrison[1-4]

1. Department of Pharmaceutical Sciences, College of Pharmacy, University of Nebraska Medical Center, 985830 Nebraska Medical Center, Omaha, NE 68198, United States

2. Center for Drug Delivery and Nanomedicine, University of Nebraska Medical Center, 985830 Nebraska Medical Center, Omaha, NE 68198, United States

3. Eppley Cancer Center, University of Nebraska Medical Center, 985950 Nebraska Medical Center, Omaha, NE 68198, United States

4. Department of Biochemistry and Molecular Biology, College of Medicine, University of Nebraska Medical Center, 985870 Nebraska Medical Center, Omaha, NE 68198, United States

5. Department of Pathology and Microbiology, University of Nebraska Medical Center, Omaha, NE, United States

6. Department of Radiology, Duke University Medical Center, Durham, NC, United States

Supplementary contents

Materials and methods **2**

Table S1. Biodistribution Studies in Tumor-Bearing SCID Mice **6**

Figure S1. Correlation of in vitro bioluminescence of PC-3-Luc vs cell numbers **8**

Figure S2. Stability study of ^{177}Lu-DOTA SP714 in PBS, ascorbic acid and selenomethionine........ **7**

Figure S3. Representative images of PC-3-Luc inoculated mice, radiance changes of tumor and tumor growth of models **10**

Figure S4. Correlation of tumor uptake of ^{177}Lu-DOTA SP714 and blood perfusion agents in animal models........ **10**

Figure S5. Body weight changes of PC-3 and PC-3-Luc implanted mouse model **9**

Figure S6. Representative confocal microscopy images of adjacent tumor sections with Hoechst perfusion, pimonidazole staining and autoradiography in orthotopic mice model........ **11**

Figure S7. Representative confocal microscopy images of adjacent tumor sections with Hoechst perfusion, pimonidazole and autoradiography staining in orthotopic mice model........ **12**

Materials

Reagents: All solvents utilized for synthesis were ACS grade, purchased from Fisher Scientific and used without further purification unless otherwise stated. Acetonitrile utilized for HPLC analysis and purification was HPLC grade and purchased from Fisher Scientific. Formic acid, N,N-diisopropylethylamine (DIEA), N,N-dimethylformamide (DMF), dichloromethane (DCM), N,N'-dicyclohexylcarbodiimide (DCC), N-methylpyrrolidone (NMP), thioanisole, sodium hydroxide, L-ascorbic acid, 4-(2-hydroxyethyl)-1-piperazineethanesulfonic acid (HEPES), bovine serum albumin (BSA), Hoechst 33342 and O.C.T. Compound were purchased from Fisher Scientific (U.S.). Water was deionized by Millipore® Milli Q Biocell Ultrapure Water System before use. D-Luciferin, O-Benzotriazole-N,N,N',N'-tetramethyl-uronium-hexafluoro-phosphate (HBTU), Fmoc-protected natural amino acids and the appropriate Rink Amide resins were purchased from Nova Biochem (U.S.). Ham's F-12K (Kaighn's) medium, phosphate buffered saline (PBS), and mammalian protein extraction reagent (M-PER) were purchased from Thermo Scientific (U.S.). Naturally abundant lutetium chloride ($^{nat}LuCl_3$), triisopropyl silane, trimethylsilyl chloride, hydrochloric acid (HCl) and 3,6-dioxa-1,8-octanedithiol were purchased from Sigma-Aldrich (U.S.). Radioactive lutetium chloride ($^{177}LuCl_3$) was purchased from the National Isotope Development Center operated by the United States Department of Energy, Office of Science. Sodium Pertechnetate ([^{99m}Tc]$NaTcO_4$) was purchased from Cardinal Health™. The human prostate cancer PC-3 cell line was obtained from American Type Culture Collection (U.S.) and cultured under vendor recommended conditions. TrypLE™ Express was purchased from Invitrogen (U.S.). Transdux™ was purchased from System Biosciences. Pre-made lentiviral expression particles were purchased from AMS Biotechnology Limited. Pimonidazole hydrochloride and FITC Mab were purchased from Hypoxyprobe™.

Animals: Four weeks-old NOD SCID (NOD.CB17-Prkdc^{scid}/NCrCrl) mice were obtained from the Jackson Laboratory (Bar Harbor, ME). Food and water were given ad libitum. Five mice per group were kept in the same cage with an air filter cover and a light- (12 h light/dark cycle) and temperature-controlled (22±1°C) environment prior to experiments. On the day of the experiment, each animal was isolated under the same housing conditions. The animals were treated in accordance with the Principles of Animal Care outlined by the National Institutes of Health and approved by the Institutional Animal Care and Use Committee of the University of Nebraska Medical Center.

Instrumentation: The peptide was synthesized on a Liberty microwave peptide synthesizer from CEM (U.S.). A Waters e2695 system equipped with a Waters 2489 absorption detector and a Waters Q-tof Micro electrospray ionization mass spectrometer was used to perform high-performance liquid chromatography/mass spectrometry analyses. A Phenomenex Jupiter C12 Proteo 250×10 mm semipreparative column was used for the purification of bulk amounts of the BB2r-targeted peptide. The peptide was concentrated on a Centrivap (U.S.) Centrifugal Concentrator. Evaluation and purification of the radiolabeled conjugate was performed on a Waters 1515 binary pump equipped with a Waters 2489 absorption detector and a Bioscan (U.S.) Flow Count radiometric detector system, using a Phenomenex Jupiter C12 Proteo 250×4.6 mm column. Solid phase extraction was performed using Empore (U.S.) C18 10 mm high-performance extraction disks. Gas anesthesia was administered at a vaporizer setting of 5% isoflurane (Halocarbon Corp, River Edge, NJ) with 0.5 L/min oxygen using an EZ Anesthesia apparatus (EUTHANEX Corp, Palmer, PA). Cell sorting was accomplished by fluorescence-activated cell sorting of living cells (FACS) using FACSAria. Biodistribution radiation measurements were made with a NaI (Tl) well detector constructed by AlphaSpectra, Inc. (U.S.). Bioluminescence images were acquired by IVIS® Spectrum. Animal tissue slides were generated by microtome cryostat HM 500 OM from MICROM. Confocal microscopy images were taken on a Leica LSM 510 META Microscope equipped with an argon laser and processed by ZEN black software. The autoradiography studies were accomplished by GE Lifesciences Typhoon FLA 9500 laser scanner.

Methods

Solid-Phase Peptide Synthesis (SPPS)

Peptide synthesis was performed using an automated solid-phase peptide synthesizer employing traditional Fmoc chemistry and using a Rink Amide resin. Briefly, the resin (100 µmol of the resin substituted peptide anchors) was deprotected using piperidine, resulting in the formation of a primary amine from which the C-terminus of the growing peptide was anchored. The Fmoc protected amino acids (300 µmol) with appropriate orthogonal protection were activated with HBTU and sequentially added to the resin. The resulting peptide was orthogonally deprotected and cleaved from the resin using a cocktail consisting of triisopropyl silane (0.1 mL), water (0.1 mL), 3,6-dioxa-1,8-octanedithiol (0.1 mL), trifluoroacetic acid (4.625 mL) and thioanisole (0.075 mL), respectively. The cleaved peptide was subsequently precipitated and washed using cold (0 °C) methyl-tert-butyl ether (10 mL×3). The crude conjugate was dried by a centrivap concentrator and weighed. ES-MS was used to determine the molecular mass of the prepared peptides. All conjugate were peak purified to ≥ 95% purity and quantified by RP-HPLC prior to in vitro/in vivo investigations.

HPLC Purification and Analysis Methodology

Sample analysis and radioconjugate purification were performed on an analytical Proteo column with a flow rate of 1.5 mL/min. HPLC solvents consisted of H_2O containing 0.1% formic acid (solvent A) and acetonitrile containing 0.1% formic acid (solvent B). Bulk peptide purification was performed using a semi-preparative Proteo column with a flow rate of 5.0 mL/min. For all unlabeled and $^{177/nat}$Lu-labeled conjugates, purification was achieved using an initial gradient of 80 % A : 20 % B which linearly decreased to 70 % A : 30 % B over a 15 minute time period. At the end of the run time, the column was flushed with the gradient 5 % A : 95 % B and re-equilibrated to the starting gradient.

Labeling with $^{nat}LuCl_3$

For the convenient characterization of the Lu-labeled peptide, naturally abundant ^{nat}Lu was used to substitute for ^{177}Lu in the ES-MS and in vitro binding studies. A sample of the conjugate (1 mg) was dissolved in ammonium acetate buffer (1 M, 200 µL, pH 5.5) and mixed with a solution of $^{nat}LuCl_3$ (5.5 mg). The solution was heated for 60 min at 50 °C. After cooling to room temperature, the ^{nat}Lu-conjugate was peak purified by RP-HPLC. The ^{nat}Lu-conjugate was ≥ 95% purity before mass spectrometric characterization and in vitro binding studies were performed.

Radiolabeling BBN-analog with $^{177}LuCl_3$

Radiolabeling was performed on BBN-analog by mixing 100 µg samples with 37 MBq [^{177}Lu]$LuCl_3$ in ammonium acetate buffer (1 M, 200 µL, pH 5.5). The solution was heated for 60 min at 90 °C. In order to separate radiolabeled peptide from unlabeled peptide on HPLC, 4-5 mg $CoCl_2$ was added to the crude peptide mixture and incubated for 5 min at 90 °C to increase the hydrophobicity of unlabeled conjugate. The resulting radioconjugate was allowed to cool to room temperature, peak purified using RP-HPLC (≥95%) and concentrated using a C_{18} extraction disk. Elution of the extraction disk with ethanol/sterile saline solution (6 : 4, 150 µL × 2). The ethanol eluent was evaporated by purging with nitrogen gas.

Distribution Coefficient Studies

The partition coefficient was determined for the ^{177}Lu-labeled conjugate. In a microcentrifuge tube, 0.5mL of 1-octanol was added to 0.5 mL phosphate-buffered saline (pH 7.4) containing the radiolabeled peptide (100,000 cpm). The solution was vigorously stirred for 2 min at room temperature and subsequently centrifuged (100g, 5 min) to yield two immiscible layers. Aliquots of 100 µL were taken from each layer and the radioactivity of each was quantified by a γ-counter. Statistical analysis using the unpaired two-tailed Student's t-test was performed in order to compare the logD values and p values < 0.05 were considered statistically significant.

Radiochemical Stability Studies

In a vial, 25 µg samples of ^{177}Lu-conjugate were mixed with 74 MBq of [^{177}Lu]$LuCl_3$ in an ammonium acetate buffer (0.5 M, 120 µL, pH 5.5). The mixture was heated at 90 °C for 40 min and subsequently heated for an additional 5 min in the presence of 4-5 mg $CoCl_2$. The resulting radioconjugate was peak purified using RP-HPLC and concentrated using C18 extraction disks with ethanol/sterile PBS (6:4, 200 µL). The ethanol was evaporated using nitrogen gas and the resulting solutions were diluted with either 1 mL A: PBS, B: Ascorbic acid in 0.9% sodium chloride solution (final ascorbic acid concentration, 40mg/ml), or C: Selenomethionine (Se-Met) in buffer B (final Se-Met concentration, 0.2mg/ml). The purity

of the radioconjugate solution was determined by HPLC at 24, 48 and 72 h.

In Vitro Competitive Cell-Binding Studies

The half maximum inhibitory concentration (IC_{50}) of the ^{nat}Lu conjugate was determined by competitive displacement cell-binding assays using PC-3 cells. The ^{nat}Lu-conjugate was used as substitutes for the corresponding ^{177}Lu-radioconjugate. Briefly, PC-3 cells (~3×10^4) were suspended in RPMI 1640 media (pH 7.4, 4.8 mg/mL HEPES, and 2 mg/mL BSA) and incubated at 4°C for 40 min in the presence of radiolabeled [^{125}I-Tyr_4]-Bombesin and various concentrations of the ^{nat}Lu-conjugate. At the end of the incubation period, the cells were centrifuged, aspirated and washed with media a total of three times. The cell-associated activity was measured using a γ-counter and the IC_{50} values determined by nonlinear regression using the one-binding site model of GraphPad PRISM 5 (U.S.).

Establishment of Stably Transfected PC-3-Luc Cells

To generate the PC-3-Luc cells, we utilized pre-made lentivirus particles encoding luciferase and GFP (EF1a-Luciferase (firefly)-2A-GFP (Puro), AMSBIO) for transfection. Briefly, normal PC-3 cells were seeded in a 24-well plate using 0.5 mL of medium containing 0.5×10^5 cells/mL and incubated overnight. The medium was removed and replaced with 0.5 mL of fresh medium pre-mixed with 2.5 µL Transdux™. To each well was added 2 µL of the lentivirus particle solution and swirled to mix. The plate was returned to the incubator and cultured for 3 days. Once the cells grew to confluence, they were harvested and transferred to larger plates (from 6-well plates to 60 mm dishes). 1.5 mL of suspended cells in PBS at a concentration of 5×10^6/mL was sorted by the FACS method to give the GFP positive (PC-3-Luc) cells that were returned to culture for in vitro and in vivo studies.

Cell Culture

The normal PC-3 cells and PC-3-Luc were cultured in our laboratory, as per ATCC protocols, in Ham's F-12K medium containing 10% FBS, 2.5 mM L-glutamine, 15 mM HEPES and 0.5 mM sodium pyruvate. Cells were incubated at 37 °C at 5% CO_2.

Animal Studies

All animal experiments were performed in accordance with the NIH animal use guideline and protocol approved by the Institutional Animal Care and Use Committee (IACUC) at the University of Nebraska Medical Center (Omaha, NE).

Development of PC-3 Xenograft SCID Mice

The mice were inoculated with 5×10^6 PC-3 cells suspended in Matrigel® into the flanks. The tumor size was monitored during the growth by caliper measurement.

Development of PC-3 Orthotopic SCID mice

The PC-3-Luc cells were harvested by 5 mL TrypLE™ Express. After washing with PBS, the cells were resuspended in fresh culture medium at 0.5×10^6/mL. The single-cell suspension was mixed with Matrigel® in a 1:1 ratio. Animals was anesthetized with ketamine:xylazine (100mg/kg : 10mg/kg) via intraperitoneal injection. The abdomen area was first shaved with a trimmer, a hair removal solution applied to the trimmed area for 5 sec and the nude skin cleaned. The skin was cleaned with iodine solution and a one cm midline incision was made. After separated from the skin, muscles were cut and the prostate gland was exposed. 50 µL cell suspension was injected into a dorsal prostatic lobe using a 25-gauge needle (EXELINT®, zero dead space). The wound was closed in two layers with Henry Schein® Sorbocryl™ 4/0 suture and wound clips. Animals were given analgesic drugs every 12 h for 3 days. The clips were removed at 14 days post-surgery. The animals were monitored continuously for the following weeks.

In Vivo Biodistribution Studies

The body weight, tumor volume and tumor luminescence of the mice were recorded every three days. The mice were divided into three groups based on tumor volume (<300, 300-700 and >700 mm^3). Each mouse (average weight: 20 g for female mice and 25g for male mice) was treated with pimonidazole solution (80mg/kg in PBS) via intraperitoneal injection. After 1h, the mice received an intravenous injection of 10 µCi (370 kBq) of the radio-RP-HPLC peak purified ^{177}Lu-labeled conjugate (^{177}Lu-DOTASP714) in 100 µL of PBS. After an additional 1h, the mice were injected intravenously with a PBS

solution containing 10 μCi (370 kBq) of [^{99m}Tc]$NaTcO_4$ and 15mg/kg Hoechst 33342. The animals were sacrificed 5 minutes later, and their tissues collected. The excised tissues were weighed, the radioactivity in each tissue was measured, and the %ID/g was calculated for each tissue. Due to the overlap of the energy spectra of Technetium-99m and Lutetium-177, we first measured the total radioactivity in each organ on day 1 (A) and measured again at day 4 (B). Since the Technetium-99m had completely decayed by day 4, the radioactivity observed at time point B was Lutetium-177. A decay correction was performed to calculate the amount of radioactivity associated with the Lutetium-177 component on day 1 (C). The difference of value between A and C was the radioactivity of Technetium-99m at day 1.

Bioluminescent Imaging

Bioluminescent imaging was acquired according to standard protocol from the UNMC Small Animal Imaging Core Facility. Imaging and quantification of the signals were performed by Living Image® 4.5 software (PerkinElmer® Health Sciences). For in vitro imaging, PC-3-Luc cells were diluted and plated in a black, clear bottom 96-well plate. 50 μL D-luciferin (150 μg/mL) in media was added to each well 5 min prior to imaging. For in vivo imaging, mice were given 100 μL D-luciferin (150 mg/kg) 15-20 min prior to imaging and anesthetized by isoflurane. Mice were transferred to the imaging box and anesthesia nose cones attached. Images were acquired with auto exposure. Regions of interest from each image were selected and quantified.

Microscopy and Autoradiography

The tumors from mice were washed by deionized water, dried and embedded by O.C.T compound on dry ice. The frozen tumor sections were cut at 10 µm and stored at -80 °C for the following studies. The adjacent tumor slides were scanned for Hoechst 33342 and pimonidazole by confocal microscopy and exposed to storage phosphor screens (BAS-IP SR 2025 E, GE Healthcare) for 3 days. The phosphor screens were subsequently scanned by Typhoon FLA 9500 (GE Healthcare) with 25 µm pixel size. The range of interest (ROI) was drawn to exclude the edge of each tumor slide. The fluorescent arithmetic mean intensity of images were measured by Zeiss ZEN (blue edition) software.

Table S1. Biodistribution Studies in Tumor-Bearing SCID Mice.

Tissue (%ID/g)*	^{177}Lu-BB2r conjugate uptake			Perfusion ([^{99m}Tc]TcO_4^{-})		
	Tumor volume (mm^3)					
	<300	300-700	>700	<300	300-700	>700
	**Subcutaneous**					
**blood**	0.49 ± 0.40	0.78 ± 0.81	2.27 ± 0.60	1.01 ± 0.06	0.88 ± 0.34	2.61 ± 1.54
**heart**	0.16 ± 0.05	0.16 ± 0.03	0.68 ± 0.48	0.40 ± 0.24	0.22 ± 0.11	0.43 ± 0.38
**lung**	1.08 ± 1.55	0.64 ± 0.39	1.39 ± 0.67	0.74 ± 0.27	0.49 ± 0.21	1.24 ± 1.00
**liver**	0.29 ± 0.12	0.31 ± 0.06	0.90 ± 0.49	0.67 ± 0.43	0.41 ± 0.19	0.85 ± 0.77
**pancreas**	19.38 ± 12.99	23.82 ± 8.43	22.64 ± 7.83	2.26 ± 1.95	3.30 ± 2.30	6.22 ± 6.53
**stomach**	0.94 ± 0.53	1.68 ± 0.85	4.89 ± 3.52	2.26 ± 1.06	2.41 ± 1.25	3.03 ± 2.52
**spleen**	1.46 ± 1.32	2.53 ± 1.31	3.02 ± 0.63	1.35 ± 1.21	0.87 ± 0.74	0.43 ± 0.51
**small intestine**	2.53 ± 1.39	2.82 ± 1.14	4.21 ± 1.92	0.52 ± 0.38	0.61 ± 0.43	0.53 ± 0.43
**large intestine**	1.38 ± 0.75	2.63 ± 2.12	3.75 ± 2.89	0.44 ± 0.35	0.43 ± 0.47	0.53 ± 0.53
**kidney**	2.75± 1.29	2.59 ± 1.02	6.64 ± 2.83	0.66 ± 0.33	0.53 ± 0.19	1.11 ± 0.92
**tumor**	4.16 ± 1.74	5.35 ± 1.75	9.44 ± 3.66	0.51 ± 0.31	0.68 ± 0.49	0.86 ± 0.35
**muscle**	0.17 ± 0.12	0.15 ± 0.07	0.46 ± 0.24	0.19 ± 0.11	0.11 ± 0.04	0.21 ± 0.20
**bone**	0.20 ± 0.12	0.26 ± 0.19	0.67 ± 0.07	0.34 ± 0.27	0.16 ± 0.06	0.34 ± 0.31
**brain**	0.03 ± 0.02	0.03 ± 0.01	0.09 ± 0.05	0.06 ± 0.04	0.03 ± 0.02	0.06 ± 0.05
**excretion† (%ID)**	68.51 ± 23.40	73.16 ± 7.00	59.05 ± 12.41	83.64 ± 9.63	85.78 ± 9.65	82.76 ± 12.78
	**Orthotopic**					
**blood**	0.27 ± 0.19	0.33 ± 0.14	0.57 ± 0.12	3.13 ± 1.02	3.51 ± 1.28	3.96 ± 0.90
**heart**	0.13 ± 0.09	0.39 ± 0.49	0.30 ± 0.17	1.29 ± 0.61	1.20 ± 0.63	1.79 ± 0.87
**lung**	0.82 ± 0.50	0.92 ± 0.41	1.28 ± 0.47	2.86 ± 1.06	2.78 ± 1.27	3.34 ± 0.84
**liver**	0.26 ± 0.07	0.40 ± 0.18	0.52 ± 0.19	2.51 ± 0.73	2.92 ± 0.95	3.02 ± 0.77
**pancreas**	30.01 ± 12.48	40.12 ± 11.47	37.24 ± 7.29	4.94 ± 4.84	5.17 ± 5.32	4.03 ± 3.24
**stomach**	1.07 ± 0.53	1.59 ± 0.90	1.46 ± 0.62	16.33 ± 4.08	19.15 ± 6.40	15.44 ± 3.98
**spleen**	1.77 ± 1.19	2.02 ± 3.14	2.91 ± 4.89	2.84 ± 1.87	1.70 ± 1.20	1.80 ± 1.37
**small intestine**	2.97 ± 0.66	3.74 ± 1.15	3.80 ± 1.46	1.31 ± 0.31	1.36 ± 0.45	1.32 ± 0.78
**large intestine**	2.06 ± 1.02	4.52 ± 1.31	4.07 ± 1.91	1.52 ± 0.36	2.24 ± 0.57	2.14 ± 0.55
**kidney**	3.40 ± 0.92	4.80 ± 4.92	5.98 ± 3.08	1.85 ± 0.91	2.25 ± 2.25	1.45 ± 0.64
**tumor**	8.07 ± 3.23	8.55 ± 2.65	11.13 ± 2.88	2.23 ± 1.09	2.94 ± 0.76	3.19 ± 0.66
**muscle**	0.23 ± 0.12	0.22 ± 0.10	0.48 ± 0.34	0.47 ± 0.20	0.59 ± 0.18	0.63 ± 0.24
**bone**	0.29 ± 0.22	0.46 ± 0.22	0.53 ± 0.24	1.10 ± 0.73	1.27 ± 0.68	0.85 ± 0.49
**brain**	0.03 ± 0.01	0.06 ± 0.03	0.06 ± 0.02	0.14 ± 0.04	0.18 ± 0.18	0.16 ± 0.07
**excretion† (%ID)**	60.77 ± 29.28	67.22 ± 8.53	60.18 ± 10.77	39.51 ± 22.40	37.55 ± 14.08	46.43 ± 13.26

*****. Organ uptake values expressed as %ID/g and values are n ≥ 8 unless otherwise noted.

†. Excretion values (%ID) were calculated using the activity values associated with the excreted urine and fecal material contents at the time of sacrifice.

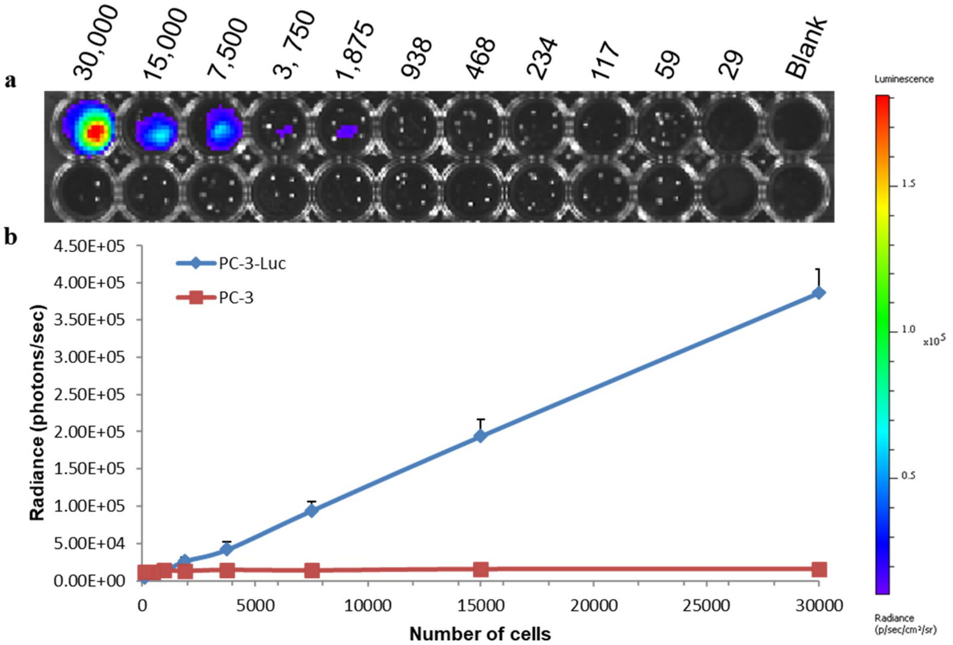

Figure S1. a) Representative images of in vitro bioluminescence of human prostate cancer cell line with or without luciferase expression gene (PC-3-Luc and PC-3). **b)** Correlation between cell number and mean radiance (photons/sec) per well (R^2=0.9992).

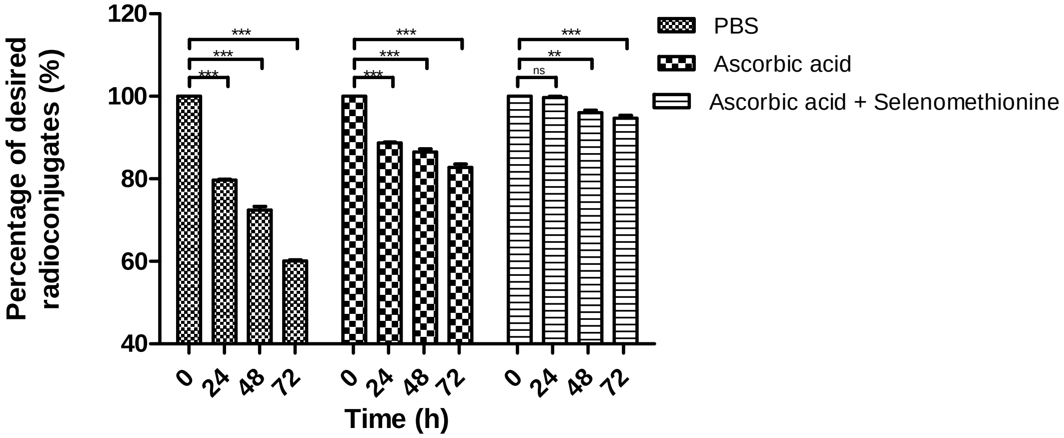

Figure S2. Stability study of [^{177}Lu]Lu-DOTA-SP714 in PBS, ascorbic acid and ascorbic acid/selenomethionine.

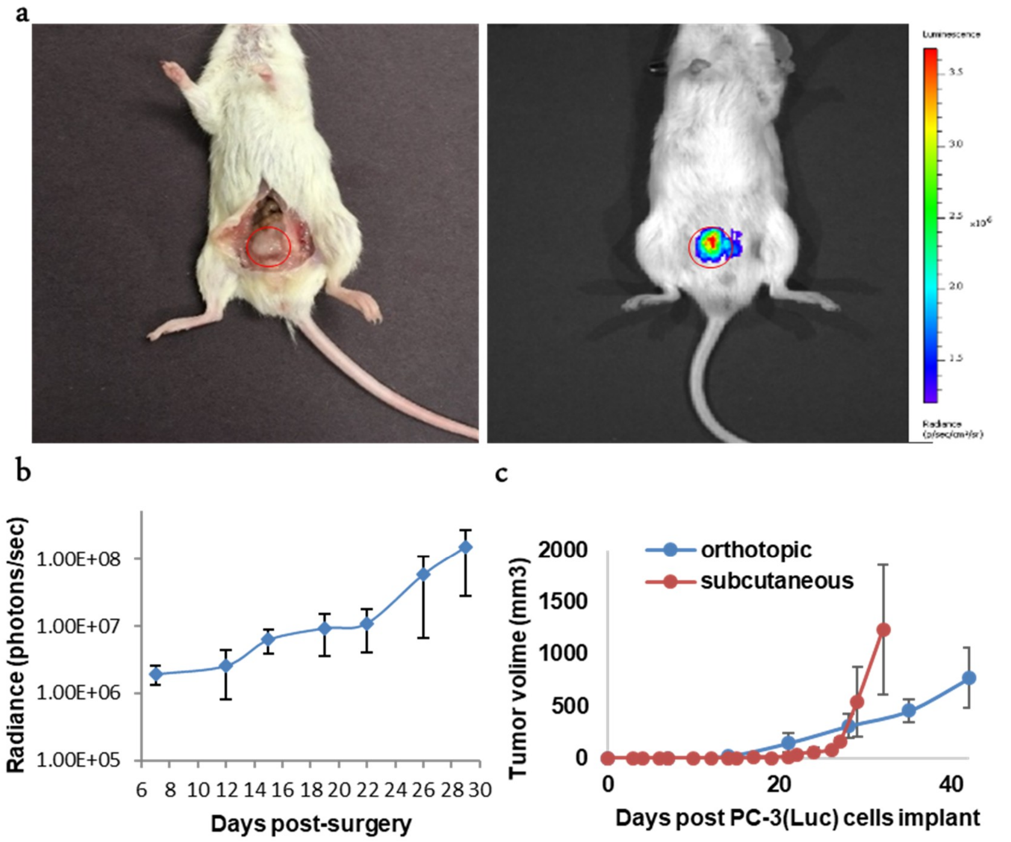

Figure S3. a) Representative images of PC-3-Luc tumors resulting from orthotopic mouse model. Circled tumor area by IVIS image system matched the actual tumor size after the dissection of mice. **b)** Mean radiance signal for the orthotopic tumors post PC-3-Luc cell implantation determined by IVIS. **c)** Mean tumor volume after PC-3/PC-3-Luc cells implantations. The tumor volume was measured by caliper and IVIS® Spectrum software in subcutaneous and orthotopic mice, respectively (Mean ± SD).

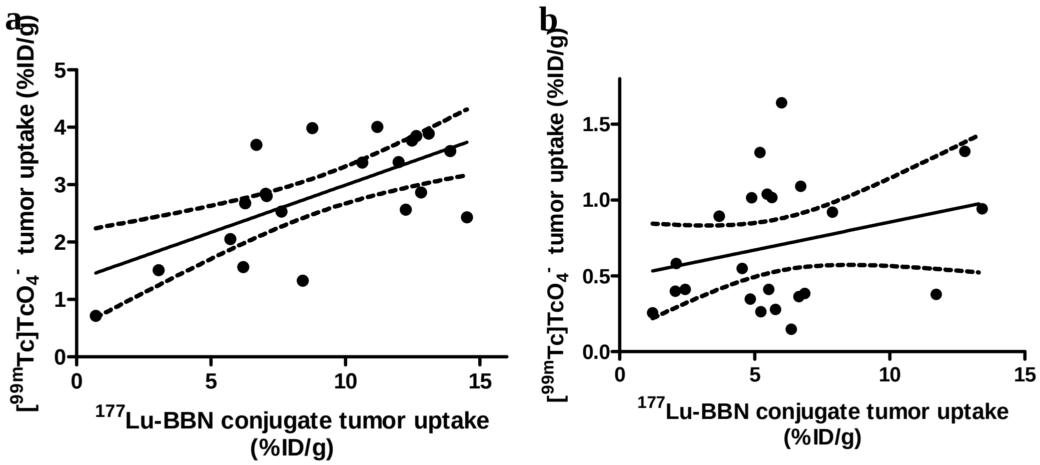

Figure S4. Correlation of tumor uptake of [^{177}Lu]Lu-DOTA-SP714 and blood perfusion agent [^{99m}Tc]TcO_4^- in a) orthotopic (n=23) and b) subcutaneous (n=24) mice model respectively.

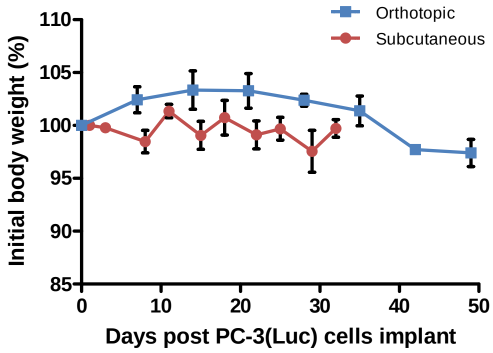

Figure S5. Body weight changes of mice after the orthotropic (blue) and subcutaneous (red) xenograft implantation.).

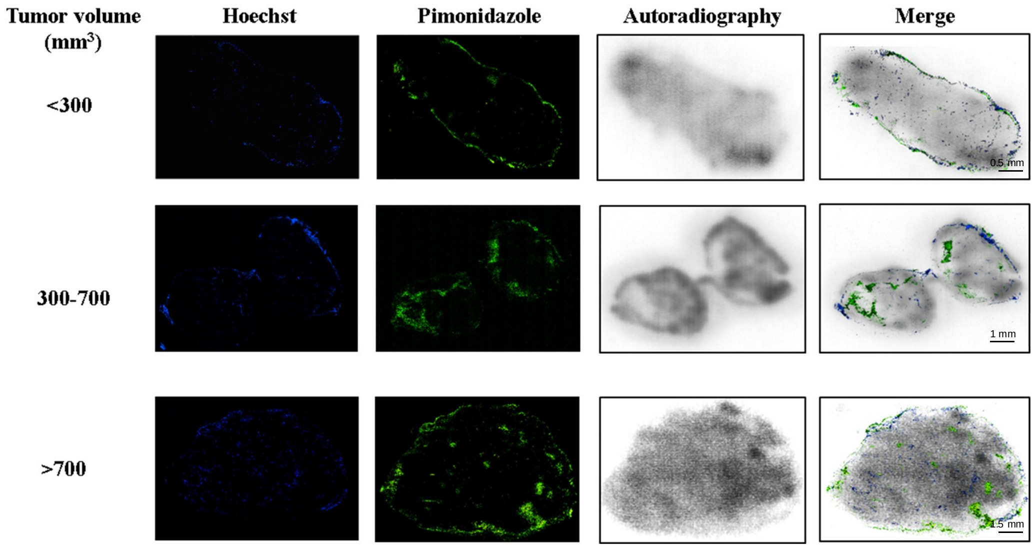

Figure S6. Representative confocal microscopy images of adjacent subcutaneous tumor sections in different tumor volume group. The sections were scanned for Hoechst 33342 (blue), the fluorescent antibody of pimonidazole (green) and underwent autoradiography. The contrast of each image was optimized for clarity.

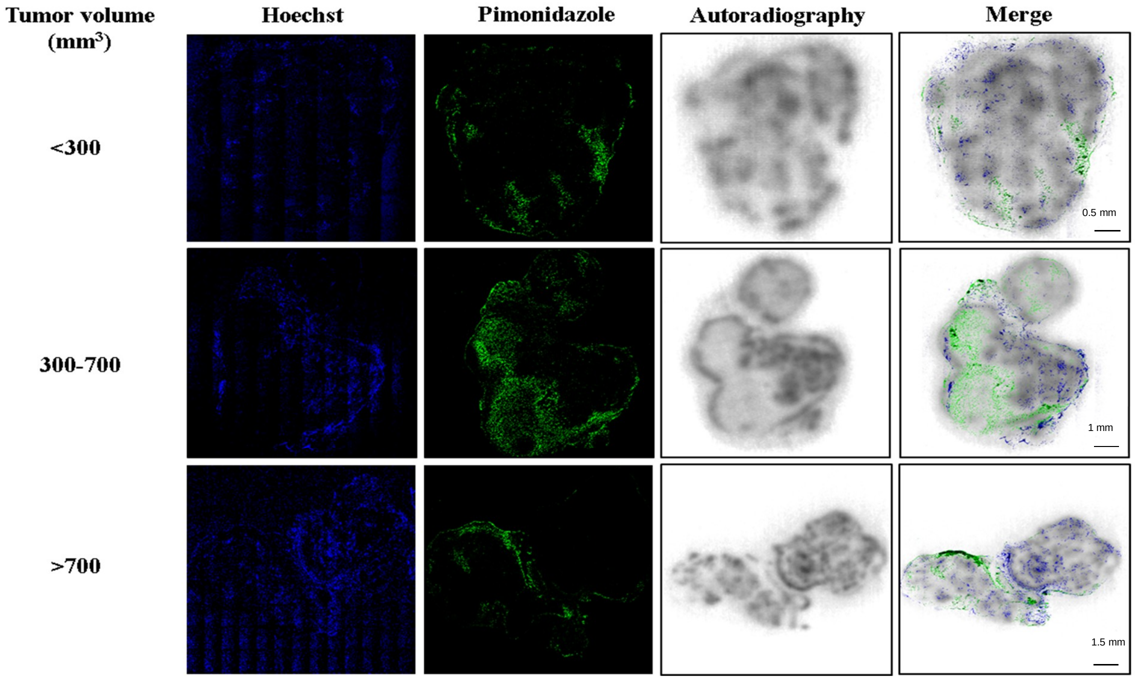

Figure S7. Representative confocal microscopy images of adjacent orthotopic tumor sections in different tumor volume groups. The sections were scanned for Hoechst 33342 (blue), the fluorescent antibody of pimonidazole (green) and underwent autoradiography. The contrast of each image was optimized for clarity.
